# Emerging roles of extracellular vesicles in the adaptive response of tumour cells to microenvironmental stress

**DOI:** 10.3402/jev.v2i0.20304

**Published:** 2013-03-05

**Authors:** Paulina Kucharzewska, Mattias Belting

**Affiliations:** 1 Section of Oncology, Department of Clinical Sciences, Lund University, Lund, Sweden; 2 Department of Oncology, Skåne University Hospital, Lund, Sweden

**Keywords:** extracellular vesicles, exosomes, shed microvesicles, cancer, tumour microenvironment, stress response

## Abstract

Cells are constantly subjected to various types of endogenous and exogenous stressful stimuli, which can cause serious and even permanent damage. The ability of a cell to sense and adapt to environmental alterations is thus vital to maintain tissue homeostasis during development and adult life. Here, we review some of the major phenotypic characteristics of the hostile tumour microenvironment and the emerging roles of extracellular vesicles in these events.

It has become widely accepted that aberrant cellular stress responses may underlie a variety of pathological conditions, including cancer (1, 2). To overcome the harsh microenvironmental barriers, tumour cells activate stress response mechanisms, which in concert with resistance mechanisms to programmed cell death confer them growth advantage and drive tumour progression. In this context, extracellular vesicles (EVs) are of particular interest as they may constitute a novel, adaptive mechanism against stressful conditions of the tumour microenvironment (3, 4). We will initially describe the complexity of the tumour microenvironment, and then discuss the potential role of EVs as mediators of tumour progression through adaptive effects to counteract microenvironmental stressors.

## The tumour microenvironment

**Fig. 1:**
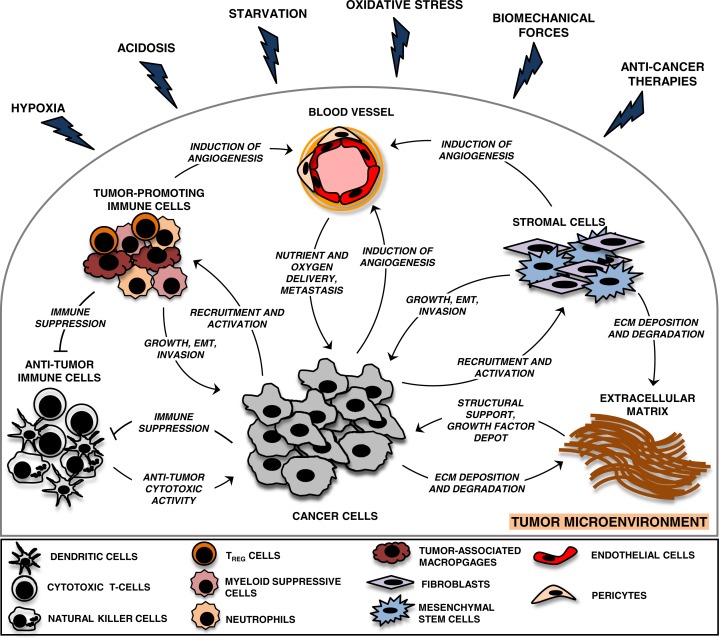
Heterotypic cellular interactions in the tumour microenvironment. The tumour microenvironment is a complex scaffold of an extracellular matrix (ECM) and various cell types. In addition to malignant cells, vascular cells, stromal cells and immune cells are common cellular residents of the tumour niche. Tumour cells mould this environment for their own needs via intercellular communication pathways, such as direct cell-to-cell contacts and the release of growth factors, matrix metalloproteases, ECM proteins and extracellular vesicles (EVs). Tumour cell-mediated stromal modifications include: suppression of anti-tumoural immune responses, deposition and degradation of ECM components, induction of vascular network formation and recruitment of stromal cells and tumour-promoting immune cells. In turn, heterogeneous tumour microenvironmental components create a favourable environment for tumour growth and dissemination. Various tumour microenvironmental stressors are inherent features of solid tumours that profoundly modify the tumour milieu and accelerate tumour progression towards malignancy.

It is well-established that cancer cells do not exist in isolation but rather within a complex milieu, known as the tumour microenvironment. This intricate niche consists of multiple cell types immersed in an extracellular matrix (ECM), and it plays a fundamental role in tumour progression, as originally proposed by Paget's “seed and soil” hypothesis (5, 6) (Fig. 1). During the course of tumour development, neoplastic cells actively recruit normal cells into their neighbourhood, which support malignant progression in multiple ways. In this context, endothelial cells and pericytes are of great importance being responsible for the formation and function of the tumour vasculature (7); fibroblasts that deposit an ECM and secrete both matrix-degrading enzymes and soluble growth factors (8); and cells of the immune system, which may provide an immunosuppressive and growth-promoting compartment (9). The three dimensional organisation and architecture of a tumour mass are provided by the ECM, which in contrast to normal matrix, is typically enriched in several proteins, such as type I collagen and heavily glycosylated glycoproteins, for example, proteoglycans. In addition, the tumour stroma regulates cellular signalling and acts as a reservoir of growth factors (10). The successful expansion of malignant tumours requires an active collaboration between malignant and stromal cells via heterotypic cellular interactions. Accordingly, malignant cells and subsidiary stromal cells communicate and exchange information by direct cell-to-cell contacts as well as the release of signalling molecules, such as soluble growth factors, ECM proteins (11) and the only recently appreciated EVs (3).

The driving forces of tumour microenvironmental evolution are genetic instability of malignant cells and environmental selection forces, which include endogenous, tumour-growth-induced stress stimuli, such as hypoxia, acidosis, starvation, oxidative stress, biomechanical stress and immunoediting as well as exogenous stresses, for example, therapeutic interventions (12). Together, these factors select for tumour cells that acquire intrinsic and extrinsic properties to overcome microenvironmental threats and progress (13–23). Interestingly, graded and local distribution of microenvironmental stresses in the tumour mass contributes to another feature of cancer, that is, intra-tumoral heterogeneity, which represents a major hurdle for successful large-scale tumour molecular profiling and treatment of cancer patients (24).

## EVs in cancer

EVs provide a relatively new route of communication between cancer cells and various stromal cells infiltrating the tumour interstitium. Recently, numerous studies have shown that EVs affect several stages of tumour progression, including angiogenesis, escape from immune surveillance, ECM degradation and metastasis. As comprehensive reviews of EV contribution to tumour development are provided elsewhere (3, 25, 26), a brief overview is given below.

The primary association of EVs with cancer was noticed already in the late 1970s (27). Since then, multiple studies have shown that the number of circulating EVs is increased in patients with cancer and may correlate with poor prognosis (28, 29). Tumours are characterised by the secretion of various forms of EVs, which based on the mechanism of formation can be divided into exosomes and shed microvesicles (SMVs) (25). Exosomes are vesicles of size ranging from 30 to 100 nm in diameter, and are generated intracellularly as the so-called intraluminal vesicles (ILVs) within multivesicular bodies (MVBs) (30). The secretion of exosomes into the extracellular compartment results from membrane fusion of MVBs with the plasma membrane, which can be spontaneous or induced, for example, due to cell surface receptor activation (30–32). The mechanisms of assembly and sorting of exosomes are still ill-defined, but several key molecules have been shown to regulate this process, such as Rab11, Rab27, Rab35, p53, ceramide-neutral sphingomyelinase and syndecan-syntenin-Alix (33–38). Interestingly, vesicles enriched in classical markers of exosomes (CD63, CD81) have also been shown to bud from exosomal- and endosomal-protein-enriched subdomains of the plasma membrane of T- and erythroleukemia cell lines, providing further complexity to exosome biogenesis (39–41). SMVs comprise a heterogeneous population of vesicles larger than exosomes (>100 nm in diameter) that are generated by direct budding off from the plasma membrane (30). The release process seems to be controlled by calcium influx and localised cytoskeleton dynamics, and results from the outward budding of small, cytoplasmic, membrane-covered protrusions followed by detachment from the cell surface dependent on the action of ARF6 (42). Conversely to exosomes, the rate of steady state release of SMVs is generally low, except for cancer cells that seem to release them constitutively. Regulated release of SMVs is efficiently induced upon activation of cell surface receptors with biological agonists (32).

EVs are molecularly complex entities carrying lipids, soluble and transmembrane proteins, various RNA species and DNA sequences of retrotransposons (25, 32, 43). In addition, both exosomes and SMVs have been shown to enclose mitochondrial DNA (44, 45). However, this concept still remains controversial. The actual molecular composition of EVs varies depending on the mechanism of formation as well as the type and functional state of the cell of origin, for example, exosomes isolated from malignant effusions of cancer patients contain tumour specific antigens, including Her2/Neu from ovarian cancer ascites, and Mart1 from patients with melanoma (46). By carrying bioactive molecules, and facilitating their cell-to-cell transfer, tumour-associated EVs can regulate a variety of cellular events in recipient cells that significantly impact tumour progression (47–51). So far, several mechanisms of transfer of EV-associated cargo have been described. Upon release from their cell of origin, many vesicles undergo membrane rupture, leading to pericellular release of their cargo (52). Alternatively, EVs can interact with plasma membrane receptors (47, 53–56), or may reach the interior of target cells by plasma membrane fusion or through endocytosis. In these instances, the cargo molecules are released inside target cells, and thereby may interact with their signalling machinery (48–51). Interestingly, some vesicles may migrate significant distances by diffusion, and ultimately enter biological fluids, such as cerebrospinal fluid, blood, saliva and urine (26). This enables long-range exchange of EV-mediated information, which is relevant in the context of pre-metastatic niche formation (57). In addition, the abundance of tumour EVs in biological fluids offers an interesting possibility to use them as non-invasive biomarkers in the management of cancer patients (26, 28, 29).

**Fig. 2:**
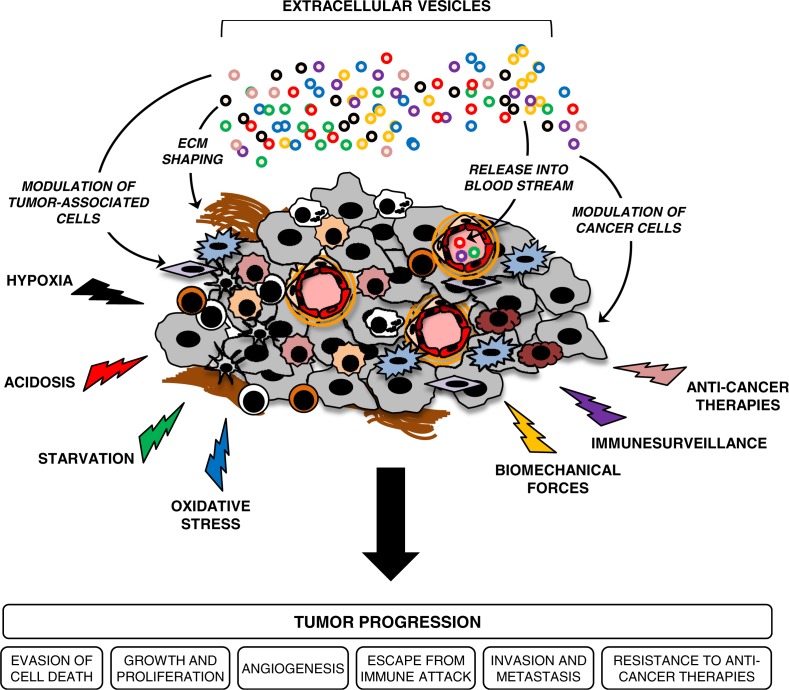
Extracellular vesicles (EVs) are potential conveyors of stress-mediated tumour progression. EVs are shed from various cellular components of the tumour milieu to mediate exchange of signalling proteins and genetic material, which altogether may support tumour growth and progression. Diverse tumour microenvironmental stress conditions augment tumour-promoting activities of EVs by modulating their secretion and trafficking in the extracellular space, as well as altering their molecular content and functional activity. Upon release, EVs may also enter the circulation and mediate long-range exchange of EV-associated cargo that may support the process of pre-metastatic niche formation. In addition, circulating EVs carrying multifaceted, molecular stress signatures may offer unique, non-invasive biomarkers that can be used in the management of cancer patients.

Given that different aspects of tumour progression are driven by stress-mediated adaptive mechanisms, it is tempting to postulate that tumours employ the EV machinery to cope with stressful conditions and to ultimately progress (Fig. 2). Below, we summarise and highlight recent findings related to this idea.

## EVs in stress-induced tumour progression

### Stress-mediated secretion and trafficking of EVs

Several reports have documented that cells vesiculate in response to different types of stresses, such as hypoxia (58–60), acidosis (61), oxidative stress (62–64), thermal stress (63, 65, 66), radiation (58), shear stress (67) and cytotoxic drugs (68). In a study by Levine and colleagues, a p53-regulated gene product, TSAP6, was shown to trigger exosome production in lung cancer cells undergoing γ-radiation (36). These *in vitro* data were lately corroborated in TSAP6 knockout mice exhibiting an impaired DNA damage-induced, p53-dependent exosome secretory pathway (69). Given that various stressors activate different signalling pathways, the existence of alternative, p53-independent mechanisms involved in stress-mediated exosome release may be anticipated. In support of this idea, a study by Trajkovic et al. showed that ceramide regulates the biogenesis and dynamics of ILVs destined for secretion as exosomes (37). Ceramide accumulation and a ceramide-mediated stress response occur as a reaction to various factors, such as lipopolysaccharide, interleukin 1β, tumour necrosis factor (TNF)-α, serum deprivation, irradiation and various cytotoxic drugs (70). Hence, it is tempting to speculate that cellular accumulation of ceramide triggers increased production and secretion of exosomes as an adaptation to stressful conditions. Further studies are needed to test this possibility. Interestingly, p53 has been shown to modulate intracellular ceramide levels through generation of O_2_^−^ in glioma cells (71), indicating that the p53 signalling pathway may additionally stimulate exosome release in a ceramide-dependent manner.

Emerging findings suggest that oncogenes, such as epidermal growth factor receptor (EGFR) or its mutant, EGFR variant III (EGFRvIII), hypoxia-inducible factor (HIF)-1α and K-ras may trigger the release of EVs from cancer cells (49, 60, 72, 73). While HIF-1α has been shown to mediate hypoxia-dependent secretion of exosomes in breast cancer cells (60), the contribution of EGFR and K-ras to stress-mediated cellular vesiculation is unknown. A recent report by Wang and colleagues showing that hypoxia activates EGFR through HIF-dependent formation of caveolae, provides a potential mechanism for HIF-mediated vesiculation in response to hypoxia (74). Further studies are required to verify whether a CAV1/EGFR signalling axis and/or other components of the HIF signalling pathway regulate EV secretion.

The tumour-promoting activities of EVs are highly dependent on efficient transfer to recipient cells, as described earlier in this review. Based on recent findings, stressful conditions of the tumour milieu may modulate some of the transfer mechanisms, for example, vascular endothelial growth factor (VEGF), interleukin 1β and FasL were shown to reside inside vesicles, and to evoke tumour-promoting activities only when liberated upon disruption of vesicle membrane integrity (52, 75, 76). How vesicle membrane disruption occurs *in vivo* is still ill-defined; however, Taraboletti et al. recently provided *in vitro* data showing that acidosis triggers the rupture of tumour-derived SMVs, resulting in VEGF release and enhanced endothelial cell migration (52). In addition to releasing soluble content of EVs, low pH may activate EV-associated cargo. In support of this notion, a study by Giusti et al. suggested that ovarian cancer cells release SMVs containing inactive proenzyme cathepsin B, that is, a cysteine proteinase that facilitates tumour invasion via ECM degradation (77). The tumour-promoting activities of SMV-associated cathepsin B may occur specifically in acidic compartments of the tumour milieu, as it becomes activated at low pH (77). Acidosis was further suggested to increase the uptake of tumour-derived exosomes through fusion with the plasma membrane of recipient cells. High rigidity and sphingomyelin/ganglioside GM3 content in exosomes released at low pH were likely responsible for the increased fusion efficiency (61). Collectively, these studies indicate that acidosis, which is a hallmark of the tumour microenvironment, plays a key role in modulating tumour-promoting activities of EVs by altering their activity and trafficking. The specificity of vesicular cross-talk may also be provided by stress-mediated modulation of receptor–ligand interactions. In support of this idea, hypoxic cancer cells were shown to release tissue factor (TF)/VIIa-bearing SMVs that stimulated protease-activated receptors type 2 (PAR-2) induced by hypoxia on target cells. EV-dependent PAR-2 activation resulted in increased secretion of the pro-angiogenic heparin-binding EGF-like growth factor (HB-EGF) from hypoxic endothelial cells (47).

### Microenvironmental stressors modulate the molecular composition of EVs

Extensive analyses by various techniques have partially decoded the molecular composition of EVs derived from various types of cells, including tumour cells (51, 78–81). Any phenotypic changes imposed by stressful conditions on a parental cell may affect the content and function of shed EVs. Accordingly, thermal- and oxidative stress imposed on leukaemia/lymphoma T and B cells were shown to induce the release of exosomes enriched in Natural Killer Group 2, member D (NKGD2) ligands, such as MICA/B and ULBP1 and 2, which provided them with immunosuppressive properties (63). Further, exposure of aggressive B-cell lymphoma cells to the anti-CD20 chimeric antibody rituximab resulted in the secretion of CD20-positive exosomes, which in turn protected lymphoma cells from antibody and complement-dependent cytolysis (82). Finally, treatment of cancer cells with cytotoxic drugs, radiation and hypoxia caused the release of EVs enriched in heat shock proteins (HSPs) (68), anti-apoptotic survivin (83) and pro-coagulant TF (47), respectively.

Along with the use of robust proteomic approaches, it has become evident that stress-mediated modifications of EV content are more profound than primarily anticipated. In accordance with this, hypoxic epidermoid carcinoma cells were shown to secrete numerous proteins that have the potential to modulate the tumour microenvironment, and that partly were enriched in exosomes (84). However, the significance of exosomes in mediating hypoxia-dependent angiogenesis and tumour development remains to be determined. Profound differences in the EV-associated protein cargo were observed between EVs derived from primary (SW480) and metastatic (SW620) colorectal cancer cells. Accordingly, SW480-derived EVs contained proteins involved in cell adhesion, whereas SW620 EV-enriched proteins were associated with cancer progression, invasion, metastasis and multidrug resistance (79). Since highly metastatic cell phenotypes result from cumulative effects of various stress conditions imposed on tumour cells, these data provide indirect proof that cellular stress modulates EV-associated cargo and potentially EV function. Although it was not pursued whether EVs from primary and metastatic cancer cells serve different functional goals in the metastatic process, the data provide important information for future studies addressing these questions.

Lipids constitute yet another type of EV-associated cargo that may undergo changes in response to cellular stress responses. Membrane biophysical analyses provided in a study by Parolini et al. showed that exosomes from melanoma cells cultured under acidic conditions were characterised by increased membrane rigidity as compared to vesicles from control cells (61). These changes were related to altered lipid composition, as acidic exosomes were enriched in ganglioside GM3 and sphingomyelin. As a consequence of these lipid modifications, acidic exosomes fused more efficiently with the plasma membrane of recipient cells, resulting in enhanced transfer of various signalling molecules (61).

As for lipids, few studies have addressed stress-mediated changes in RNA content of tumour-associated EVs. So far, it has been shown that breast cancer cells exposed to hypoxia secrete exosomes with increased levels of miR-210, pointing at the potential for qualitative differences between normoxic and hypoxic exosomes (60). Further, more robust miRNA analysis performed in a study by Hergenreider et al. demonstrated that endothelial cells treated with shear stress secrete exosomes enriched in miR-143 and miR-145. Interestingly, these changes in miRNA contents provided exosomes with atheroprotective properties (85). Since the discovery of mRNAs as molecular cargo of EVs (80) there has, to our knowledge, been no comprehensive analysis to elucidate how stress conditions modulate mRNA content of tumour-associated EVs. However, some indirect conclusions can be drawn from studies investigating molecular composition of EVs derived from non-malignant cells. Accordingly, oxidative stress in mast cells was shown to induce massive changes in the exosome-associated mRNA content (62). Similarly, endothelial cells secreted exosomes enriched in mRNAs and proteins specific for stress conditions imposed on donor cells (86). Overall, these results suggest that environmental stress conditions evoke alterations in the protein, lipid and RNA content of EVs. Consequently, tumour-associated vesicles acquire new biological functions in the tumour microenvironment; whether these changes promote tumour development remains to be conclusively shown.

Given that stress conditions of the tumour milieu mediate tumour progression, EV-associated molecular stress signatures may offer a great opportunity for the development of prognostic and predictive biomarkers in the management of cancer patients. In support of this concept, an increasing number of studies suggest that circulating EVs are constantly released from the tumour to reflect the dynamic nature of cancer and are accessible for repeated isolation from body fluids (28, 29, 51, 87–89).

### EVs as conveyors of stress-mediated tumour progression

According to general wisdom, hypoxic tumour cells secrete a plethora of soluble factors, for example, VEGF-A, into the extracellular space, which collectively activate endothelial cells, and thus induce hypoxia-driven tumour angiogenesis. We recently showed that hypoxic glioma cells release TF/VIIa-bearing EVs that specifically trigger up-regulated PAR-2 on hypoxic vascular endothelial cells (47). Similarly, hypoxia-mediated induction of TF-bearing SMVs was observed in ovarian cancer cells, indicating that tumour EVs are vehicles of TF-dependent tumour progression through clotting-dependent and independent mechanisms in the hypoxic tumour niche (90, 91). As described above, hypoxic breast cancer cells were shown to secrete exosomes enriched in miR-210 (60). Given that miR-210 is a well-established target of HIF signalling and plays important roles in the regulation of cell growth, angiogenesis and apoptosis, exosome-mediated transfer of miR-210 within the tumour milieu may contribute to hypoxia-driven tumour progression (92).

Stromal cells similarly to cancer cells may respond to stress-related conditions within the tumour microenvironment by secretion of EVs, that is, mesenchymal stem cells stimulated by hypoxia were shown to release microvesicles with angiogenic effects (59). Further, both biomechanical forces and oxidative stress were shown to trigger the secretion of pro-coagulant SMVs from platelets (67, 93). Cancer-associated hypercoagulability and its tumour-promoting activities may thus be triggered by EVs secreted from cancer cells as well as auxiliary cells in response to phenotypic characteristics of the tumour microenvironment. Finally, oxidative stress seems to significantly enhance the release of exosome-associated HSP70 from arterial endothelial cells, which in turn activates monocyte adhesion to the endothelium. Hence, it may be speculated that stress-induced secretion of HSP70-bearing exosomes from tumour-associated endothelial cells could stimulate monocyte recruitment to tumours (64).

Accumulating data suggest a link between stress conditions of the tumour milieu and immunological tolerance of tumours (94). However, the mechanisms underlying this process are still ill-defined. Cancer cells may employ vesiculation as a strategy to efficiently blunt immune surveillance mechanisms, and survive in this hostile environment (32). Interestingly, a recent study by Hedlund et al. suggests that oxidative stress imposed on tumour cells triggers the release of NKG2DL-expressing tumour exosomes, which mediate tumour escape from cytotoxic immune attack (63). Moreover, tumour cells may evade complement-induced lysis by SMV-mediated shedding of terminal components of complement from the plasma membrane (95). This mechanism, called “complement resistance”, may provide protection of tumour cells from antibody-mediated immune attack. Exosomes from various cancer cells were shown to expose Fas ligand (FasL, CD95L) of the death receptor Fas (CD95), which induces T-cell apoptosis and attenuates the function of adaptive immune cells (96, 97). Tumour-associated EVs may also promote the function of regulatory T (T_Reg_) cells (98, 99), impair natural cytotoxic responses mediated by natural killer cells (63, 100), down-regulate dendritic cell differentiation from monocytes (101) and instead turn these cells into myeloid immunosuppressive cells (101, 102). Finally, cancer cells can fuse with EVs derived from non-cancer cells, for example, platelets, thereby receiving lipids and transmembrane proteins allowing escape from immune system attack (103).

Another interesting area of research is related to the potential role of EVs in tumour resistance to various anti-cancer therapies, such as chemotherapy, immunotherapy and radiation. Recent findings suggest that chemoresistance may result from expulsion of therapeutic drugs from tumour cells through EVs. In support of this concept, cancer cells treated with doxyrubicin accumulated the drug in and released it through SMVs (104). Convincing evidence comes from a study by Safaei and colleagues, showing that exosomes released from cisplatin-resistant cells contained 2.6-fold more platinum than exosomes released from cisplatin-sensitive cells (105). Exosomes may also function to neutralise antibody-based drugs; HER2-overexpressing breast carcinoma cell lines were shown to secrete exosomes enriched in full-length HER2 protein, resulting in sequestration of the HER2 antibody Trastuzumab. As a result, the anti-proliferative activity of Trastuzumab in cancer cells was abolished (106). A similar evasion mechanism was observed for B-cell lymphoma cells treated with CD20 antibody Rituximab (82). EVs may also provide tumour cells with radiation resistance; Khan et al. showed that cervical carcinoma cells subjected to a sublethal dose of proton irradiation secreted exosomes enriched in the anti-apoptotic protein surviving (83). These data are in support of the concept that the EV pathway is involved in cancer cell self-protection under stressful conditions.

### Intercellular communication of stress via EVs

It is now well established that directly irradiated cells elicit a plethora of biological effects in neighbouring cells. This so-called radiation-induced bystander effect (RIBE) manifests in various ways, including genomic instability, a variety of damage-inducible stress responses and apoptosis (107). Interestingly, this cross-talk may be protective and non-irradiated cells can acquire properties that prepare them for possible future stresses. In support of this concept, it has been shown that human glioblastoma cells with a functional TP53 gene exhibited increased radioresistance when co-incubated with irradiated cells of the same line transfected with mutated TP53 gene, or incubated with the conditioned medium from irradiated cells. The protective effects in bystander cells exposed to the subsequent challenge were explained by nitric oxide-mediated accumulation of HSP72 and p53 protein (108). Based on a recent article by Dickey et al. the cellular machinery required to induce the RIBE might also be used to transmit signals to neighbouring cells following exposure to other forms of stress, both exogenous and endogenous (109). So far, mechanisms eliciting transfer of bystander signals involve direct cell-to-cell contact mediated by gap junctions, and indirect communication by means of soluble factors released to the extracellular space (110). In this context, EVs harbouring stress-derived molecular cargo may provide a new route of intercellular communication involved in stress-mediated bystander effect. In support of this concept, a recent study by Wang et al. demonstrated that treatment of platelets with oxidised low-density lipoproteins resulted in secretion of microvesicles, which amplified oxidative stress in recipient platelets and evoked pro-coagulant effects (93). In addition, it has been shown that mast cells exposed to oxidative stress may release exosomes with the capacity to communicate a protective signal and to induce tolerance to oxidative stress in recipient cells (62). Hence, various types of stressors may induce EV-mediated preconditioning that prepares various cells of the tumour milieu to survive and recover from the subsequent severe, otherwise lethal circumstances. This EV-mediated preconditioning effect may play important roles in tumour progression by providing resistance to various forms of stress.

## Conclusions and future directions

EVs provide an attractive signalling organelle for the demonstration of impressive functional effects in various biological systems; however, due to the complexity and heterogeneity of EV composition, deciphering the exact mechanisms behind functional data poses a great challenge. Future research is clearly warranted to understand how hypoxia and other microenvironmental stressors affect EV trafficking in the tumour microenvironment and how stress-mediated changes of recipient cells modulate their responsiveness to EVs. Such studies should significantly advance our general understanding of tumour biology and provide novel therapeutic strategies in the fight against cancer.
